# An Improved, Dual-Direction, Promoter-Driven, Reverse Genetics System for the Infectious Bursal Disease Virus (IBDV)

**DOI:** 10.3390/v14071396

**Published:** 2022-06-27

**Authors:** Xifeng Hu, Zheng Chen, Xiangdong Wu, Zhen Ding, Qinghua Zeng, Huansheng Wu

**Affiliations:** 1 Department of Preventive Veterinary Medicine, College of Animal Science and Technology, Jiangxi Agricultural University, Zhimin Street, Qingshan Lake, Nanchang 330045, China; q1159527229@163.com (X.H.); chenzheng@jxau.edu.cn (Z.C.); dxywxd2006@126.com (X.W.); dingzhenhuz@jxau.edu.cn (Z.D.); linn_zeng@163.com (Q.Z.); 2 Jiangxi Provincial Key Laboratory for Animal Science and Technology, College of Animal Science and Technology, Jiangxi Agricultural University, Nanchang 330045, China

**Keywords:** IBDV, dual-direction promoter, virus rescue, Arg186, methylation

## Abstract

The infectious bursal disease virus (IBDV), one member of the *Birnaviridae* family, causes immunosuppression in young chickens by damaging the mature B cells of the bursa of Fabricius (BF), the central immune system of young chickens. The genome of IBDV is a bisegmented, double-strand RNA (dsRNA). Reverse genetics systems for IBDV allow the generation of genetically manipulated infectious virus via transfected plasmid DNA, encoding the two genomic viral RNA segments as well as major viral proteins. For this purpose, the minus-sense of both segment A and segment B are inserted into vectors between the polymerase I promoter and the corresponding terminator I. These plasmids facilitate the transcription of the viral minus-sense genome but copy the plus-sense genome as well viral protein translation depends on the activity of VP1 and VP3, when transfected into 293T cells. To further improve rescue efficiency, dual-direction promoters were generated based on the polymerase II promoter in the reverse direction in the backbone of the pCDNA3.0 vector. Therefore, the polymerase I promoter transcribes the viral minus-sense genome in the forward direction and the polymerase II promoter transcribes viral mRNA, translated into viral proteins that produce infectious IBDV. We also found that the rescue efficiency of transfecting two plasmids is significantly higher than that of transfecting four plasmids. In addition, this dual-direction promoter rescue system was used to generate R186A mutant IBDV since Arg186 is the arginine monomer-methylation site identified by LC–MS. Our data furtherly showed that the Arg186 monomer methylation mutant was due to a reduction in VP1 polymerase activity as well as virus replication, suggesting that the Arg186 methylation site is essential for IBDV replication.

## 1. Introduction

Infectious bursal disease (IBD) is a highly contagious viral disease that disturbs young chickens (approximately 5 weeks old) [1]. The infectious bursal disease virus (IBDV), a non-enveloped virus of the *Birnaviridae* family, is the pathogen of infectious bursal disease (IBD), which causes great economic loss in the global poultry industry [2]. IBDV is also called the killer of young chickens as infection damages immature B cells in the bursa of Fabricius (BFs) [3]. Chickens are susceptible to IBDV-induced immunosuppression, resulting in a reduced response to other vaccinations, increasing susceptibility to infection from other pathogens and increasing mortality [4,5].

Unlike other RNA viruses, the genome of IBDV contains two segments of double-stranded RNA (dsRNA), segment A and segment B [6]. The full length of segment A is approximately 3.2 kb, which consists of two partially over-lapping open reading frames (ORFs) [7]. The smaller ORF expresses the non-structural protein VP5 that is dispensable for viral replication but supports virus releasing and virus induced-apoptosis and dissemination [8]. The larger ORF encodes a poly-protein, named pVP2-VP4-VP3, which is furtherly self-cleaved by VP4 to generate mature VP2 together with several small peptides as well as both VP3 and VP4 [9,10]. The matured VP2 is the major viral structural protein, and it assembles into 260 trimers to form the outer capsid, which promotes the host to produce neutralizing antibodies [11,12]. VP3 is a part member of the viral inner capsid, and it interacts with both VP1 and viral genomic dsRNA to form a viral replication complex for supporting encapsulation as well as the genome replication of IBDV [13,14]. The replication complex is involved in viral genome replication and translation, suggesting that VP3 makes a notable contribution to VP1 polymerase activity [15,16]. There is only one ORF in segment B, approximately 2.8 kb, which encodes the RNA-dependent RNA polymerase (RdRp) VP1, which is responsible for supporting viral genome copy, translation and eventual assembly into mature virion. Mature virion contains both free VP1 and genome-conjugated protein VP1 (VPg) that bind to the 5′ end of the genomic RNA segments [3,17].

With rapid molecular operation technology improving as well as further investigation of predetermined genetic modifications in virus genomes, it is urgent to establish stable, high efficiency reverse genetics systems for generating recombinant viruses. Based on previous literature, there are several approaches to producing recombinant IBDV. The first accepted use is the T7 promoter co-driven with the hepatitis delta virus Ribozyme (HDR) which cleaves at the 5′ end of the transcriptional RNA itself which can produce the full length viral genome [18]. However, the use of this T7-based system has gradually been restricted as it depends on other cell lines that stably express T7 RNA polymerase. Since the IBDV gnome is around 6 kb divided into two segments, this indicates that it is a small genome. For small virus rescue genome, RNA polymerase II may be more suitable. Thus, scientists produced the CMV enhancer promoter-driven system together with HDR for IBDV rescue [6]. In this study, minus-sense RNA supported transcription by RNA polymerase I promoter, and it exactly terminated under the control of Terminator I, which could produce infectious IBDV when combined with transfection of both VP1 and VP3 since a VP1/VP3 complex is essential for IBDV genomic copy and translation. Meanwhile, we also used this system to assess VP1 polymerase activity. According to the avian fluenza virus rescue approaches, the RNA polymerase II promoter (CMV promoter) was further integrated into the 3′ end of Terminator I, which transcribed the mRNA of the viral RNA that directly translated viral proteins. Newly translated viral proteins (VP1 and VP3) were recruited to produce infectious IBDV. Furthermore, we found that virus titers rescued by a two-way promoter-driven system were significantly higher than an RNA polymerase I-driven system due to improved transfection efficiency. This dual-direction, promoter driven rescue system was used to generate R186A mutant IBDV since R186 was identified as the monomer-methylation site of VP1. Our data revealed that VP1 monomer methylation is critical for IBDV replication.

## 2. Materials and Methods

### 2.1. Viruses and Cells

Human embryonic kidney (HEK) 293T (referred as 293T cells), purchased from the China Center for Type Culture Collection, was cultured in Dulbecco’s Modified Eagle Medium (DMEM; Gbico, Calsbad, CA, USA) supplemented with 10% fetal bovine serum (FSP500; ExCell Bio, Montevideo, Uruguay). Chicken fibroblast line DF-1 (CRL-12203; ATCC) were also maintained in DMEM contained with 10% FBS (16000-044, Gbico, New York, NY, USA). All cell lines were cultured at 37 °C with 5% CO_2_. IBDV strain JX, isolated from chicken bursal infected with IBDV, was maintained in our laboratory and adapted to replicate efficiently in both 293T and DF-1 cells.

### 2.2. Antibodies and Reagents

Rabbit polyclonal antibodies against VP1, VP2, VP3, and VP4 were all generated from rabbit serum by immunization with corresponding purified protein, respectively. Horseradish peroxidase (HRP) labeled anti-rabbit (IgG) was purchased from KPL (Millford, MA, USA). Cell lysis buffer NP-40 (50 mM Tris [pH 7.4], 150 mM NaCl, 1% NP-40) used for western blotting was purchased from Beyotime (P0013F, Shanghai, China). Immunofluorescence secondary antibody fluorescein isothiocyanate (FITC)-labeled goat anti-rabbit antibody (A0562) was purchased from Beyotime (Shanghai, China). The Exfect Transfection Reagent (T101-01/02) was purchased from Vazyme Biotechnology (Nanjing, China).

### 2.3. Plasmid Construction, Site-Directed Mutagenesis and Transfection

The full length of both segment A and segment B of the IBDV genome were amplified by reverse transcription (RT-PCR). The pUC-mA and the pUC-mB were generated by inserting the minus-strand segment A and segment B, respectively, into the backbone of pUC57 (Generalbio Technology, Anhui, China) between the Polymerase I Promoter (Pol I Pro) and the Terminator I (Ter I). The pCMV-mA and pCMV-mB were used to generate the insertion of pUC-mA and pUC-mB, respectively, into the pCDNA3.0 backbone. Both pCDNA3.0-VP1 and pCDNA3.0-VP3 were generated by inserting a VP1 or a VP3 ORF into a pCDNA3.0 vector (Invitrogen, Waltham, MA, USA). Site-directed mutagenesis was performed as previously reported to produce mutant expression vectors. All recombinant plasmids were confirmed via Sanger sequencing. All DNA plasmids were transfected into cells using Exfect Transfection Reagent based on the protocols.

### 2.4. Flag-VP1 Purified and Liquid Chromatograph Mass Spectrum (LC–MS)

Flag tagged VP1 was transfected into 293T cells for 48 h. The cells extracted by an NP-40 buffer containing protease inhibitor were further purified by anti-Flag agarose. Purified Flag-VP1 was subjected to SDS-PAGE and subsequently visualized by Coomassie brilliant blue staining, which were excised from SDS-PAGE gel and digested in a 50 mM ammonium bicarbonate buffer, containing RapidGest overnight at 37 °C with 200 ng modified sequencing grade trypsin. The digested samples were determined using high-sensitivity liquid chromatography tandem mass spectrometry with a QE Mass Spectrometer in Applied Protein Technology (Shanghai, China).

### 2.5. Western Blotting

A western blot assay was performed as previously described [19]. Briefly, whole protein was extracted by an NP-40 buffer at 4 °C for 15 min and centrifuged at 12,000× *g* at 4 °C for 10 min. An equivalent volume of protein samples was subjected to 12% SDS-PAGE and transferred to nitrocellulose membranes (GE Healthcare, Chicago, IL, USA). The membranes were blocked by 5% milk for 30 min at room temperature and then incubated with an indicated primary antibody at 4 °C for 12 h, followed by incubating with horseradish peroxidase-conjugated (HRP) secondary antibody at room temperature for 1 h. The membranes were incubated with an enhanced chemiluminescence (ECL) reagent; the signals were imaged by Amersham ImageQuant 800 (AI800) (GE Healthcare, Chicago, IL, USA). The β-actin was detected as a loading control.

### 2.6. Virus Rescue

In our study, two methods were used to rescue IBDV: pUC-mA, pUC-mB, pCDNA3-VP1 together with pCDNA3-VP3 (four plasmids system), or both pCMV-mA and pCMV-mB (dual-direction promoter system, two plasmids) were co-transfected into 293T cells for 72 or 96 h. Whole cells were twice freeze-thawed, followed by centrifuging with 12,000× *g* for 10 min. The supernatant was transferred into fresh DF-1 cells for culturing for another 48 h after replacing fresh cultures. A specific cytopathic effect (CPE) was observed under a phase-contrast microscope (Nikon, Tokyo) for detecting the CPE was used to confirm a successful IBDV rescue.

### 2.7. Assessment of IBDV Growth in DF-1 cells

Fresh DF-1 cells were individually infected with 0.1 MOI WT IBDV, R86A IBDV, and the whole cell cultures were collected at different time points (12 h, 18 h, and 24 h) after infection. The viral titers in cell cultures were titrated using 50% tissue culture infective doses (TCID_50_), as previously described (Wu et al., 2019). Briefly, the viral solution was serially diluted 10-fold in DMEM containing 2% FBS. A 100 μL aliquot of each diluted sample was added to infect fresh DF-1 cells in 96- well plates. Tissue culture wells with a cytopathic effect were considered to be positive.

### 2.8. Statistical Analysis

All data were presented as means ± standard deviations (SDS) for each group and analyzed using GraphPad Prism 5.0. The statistical significance of differences between groups was determined using the Student *t* test. A *p* value that was less than 0.05 was recorded as statistically significant.

## 3. Results

### 3.1. Polymerase I Promoter Transcripts Minus-Sense genome of IBDV

**Figure 1:**
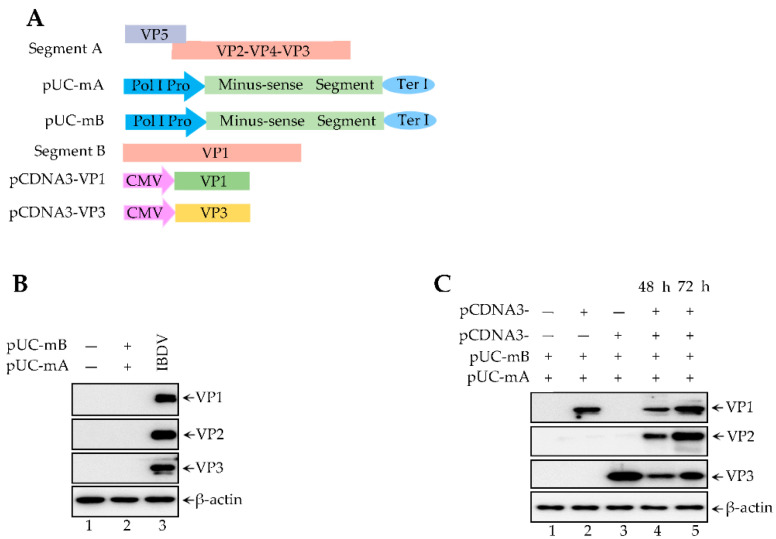
Polymerase I promoter transcripts minus-sense genome of IBDV. (**A**) Full length of minus-segment A/B were sub-cloned into the backbone of a pUC57 vector between Polymerase I Promoter and Terminator I, named as pUC57-mA and pUC57-mB. (**B**) 293T cells transfected with both pUC57-mA and pUC57-mB or infected with 1 MOI IBDV. The cell lysates were subjected to western blot using antibodies against viral protein VP1, VP2, and VP3. (**C**) Indicated plasmids were co-transfected into 293T cells at 48 h and 72 h. The cell lysates were subjected to western blot for detecting viral protein VP1, VP2, and VP3.

IBDV genome contains segment A (about 2.8 kb) and segment B (about 3.2 kb) double-strand RNA. After the virus enters the host cell, the inner virion dsRNA (viral genome) delivered into cytosol are divided into plus-sense RNA and minus-sense RNA. Both the polymerase protein VP1 and the structural protein VP3 (viral RNP complex) are recruited into plus-sense RNA and then assisted plus-sense RNA is translated into other viral proteins (VP1-VP5). Meanwhile, the RNP complex are also recruited to copy minus-sense RNA to plus-sense RNA, which is further translated into newly viral proteins. Therefore, IBDV genome transcription, replication, and translation need a viral RNP complex. Previously, reports suggested that polymerase II promoters, such as T7 and CMV promoters, can transcript the IBDV genome. The polymerase I promoter (Pol I Pro) was also used to enhance transcription of the minus-sense RNA virus, such as the avian influenza virus (AIV) and the new disease virus (NDV) since the Pol I Pro transcripts the RNA sequence with high efficiency. In this study, both minus-sense segment A and segment B were sub-cloned into a pUC57 vector between Pol I Pro and Terminator I, named as pUC-mA and pUC-mB (Ter I) (Figure 1A). The pUC-mA and the pUC-mB were co-transfected into 293T cells for 72 h. The result shown in Figure 1B reveals that both pUC-mA and pUC-mB together cannot be translated into viral proteins (Figure 1B, lane 2), indicating that uncapped viral RNA cannot itself be translated. We thus explored whether the RNP complex is required for uncapped IBDV genomic RNA translation. Co-transfection of both pUC-mA and pUC-mB together with pCDNA3-VP1 alone, pCDNA3-VP3 alone, or both of them into 293T cells for 48 h or 72 h were subjected to western blot. The western blot results in Figure 1C showed that both VP1 and VP3 co-transfection with pUC-mA and pUC-mBn (Figure 1C, lanes 4 and 5), not VP1 alone (Figure 1C, lane 2) or a VP3 alone transfection (Figure 1C, lane 3), rescued the defect translation of pUC-mA and pUC-mB. Therefore, Pol I Pro can efficiently transcript minus-sense RNA for which copy and translation required both VP1 and VP3.

### 3.2. VP1 and VP3 Are Required for Producing Infectious IBDV

**Figure 2:**
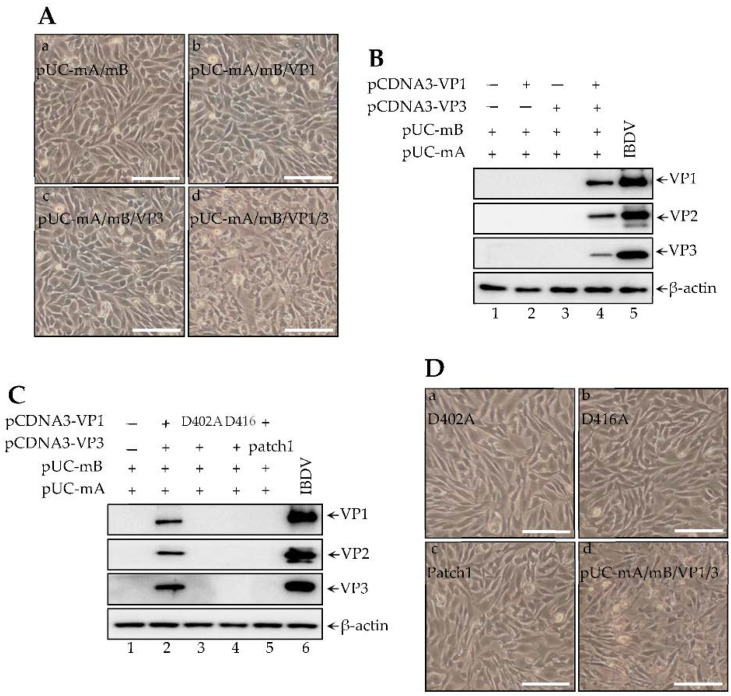
VP1 and VP3 are required for producing infectious IBDV. (**A**) Indicated plasmids were transfected into 293T cells for 72 h. Freeze thawed twice of the supernatant, which was further transferred into fresh DF-1 cells for culturing for another 48 h. A cytopathogenic effect was observed and taken imaged under the phase-phage microscopy. Scale bars, 100 μm. (**B**) Supernatant of DF-1 cells incubated with indicated supernatant of transfected 293T cells were further transferred into fresh DF-1 cells for culturing another 24 h. The cell lysates were subjected to western blot using VP1–VP3 antibodies. (**C**) Transfected 293T cells were subjected to western blot using VP1–VP3 antibodies. (**D**) Indicated plasmids were transfected into 293T cells for 72 h. Freeze thawed twice of the supernatant, which was further transferred into fresh DF-1 cells for culturing for another 48 h. A cytopathogenic effect was observed and taken imaged under the phase-phage microscopy. Scale bars, 100μm.

Since minus-sense genomic RNA copied to plus-sense RNA and then translated to viral proteins require both VP1 and VP3, we thus attempted to examine whether both VP1 and VP3 are required to produce infectious IBDV. Monolayer DF-1 cells were incubated with the supernatant of 293T cells transfected with both pUC-mA and pUC-mB together with VP1 alone, VP3 alone, or both VP1 and VP3. Only the supernatant of transfection with pUC-mA and pUC-mB together with VP1 and VP3 could cause specific cytopathogenic effects (CPE) in DF-1 cells (Figure 2Ad), instead of that transfected with other plasmids (Figure 2Aa–Ac). In addition, both VP2 and VP4, indicating infectious viral proteins, were detected in passages of pUC-mA and pUC-mB along with VP1 and VP3 transfection supernatant (Figure 2B, lane 4), WT IBDV was used as a positive control (Figure 2B, lane 5). Since VP1 polymerase activity and VP3 dsRNA binding activity are essential for initiation of IBDV replication, we thus used a similar method to evaluate the necessity of VP1 activity and VP3-dsRNA binding activity in both the viral RNA translation initiation and IBDV rescue. The viral protein VP2 and VP4, encoded by segment A, were detected in the 293T cells transfected with pUC-mA, pUC-mB, and VP3 together with VP1 (Figure 2C, lane 2), rather than with RdRp-dead mutant D402A VP1 or D416A VP1 (Figure 2C, lane 3 and lane 4). Additionally, all known VP3 dsRNA binding activity dead mutant VP3 patch1 could not promote pUC-mA translation of VP2 and VP4, even together with co-transfection with VP1 (Figure 2C, lane 5). WT IBDV was used as the positive control (Figure 2C, lane 6). The supernatant of the indicated transfection cells was subsequently seeded into fresh DF-1 cells, we found that DF-1 cells incubated with the supernatant of 293T cells transfected with pUC-mA, pUC-mB, and VP3 plus D402A VP1 (Figure 2Da), or D416A VP1(Figure 2Db), or pUC-mA, pUC-mB, and VP1 together with VP3 patch1 did not induce CPE (Figure 2Dc). Our data suggested that both VP1 and VP3 are critical for generating infectious IBDV.

### 3.3. Dual-Direction Promoter Improves Virus Rescue Efficiency

**Figure 3:**
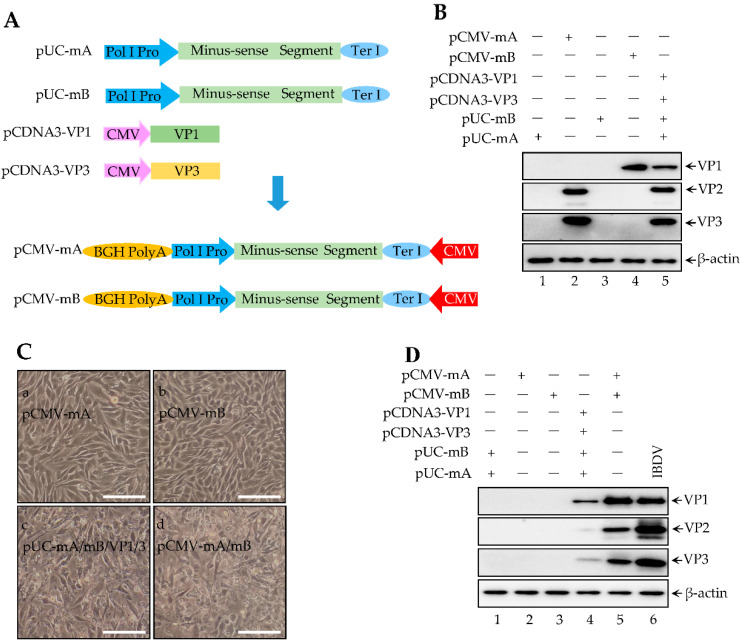

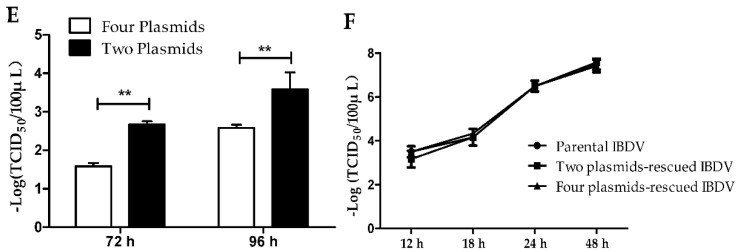
Dual-direction promoter improves virus rescue efficiency. (**A**) The model of an IBDV rescue system with four plasmids (Upper) and two plasmids (Bottom). The full length of the sequence through Poly I Pro to Ter I in the plasmid of pUC-mA and pUC-mB was inserted into the pCDN3.0 vector, named as pCMV-mA and pCMV-mB. (**B**) Indicated plasmids were co-transfected into 293T cells for 72 h. The cell lysates were subjected to western blot for detecting viral proteins, including VP1–VP3. (**C**) The supernatant of transfected cells indicated plasmids were transferred into monolayer DF-1 cells for culturing for another 48 h. CPE was observed under the phased-phage microscopy. Scale bars, 100 μm. (**D**) The supernatant of transfected cells in Figure 3C were transferred into monolayer DF-1 cells for culturing for another 24 h. Then, viral proteins were further examined by western blotting using indicated antibodies. (**E**) Supernatant of 293T cells (in 6-well plate) transfected with pUC-mA and pUC-mB plus both VP1 and VP3, or pCMV-mA and pCMV-mB for 72 h and 96 h. Collecting the whole cells for TCID_50_ to detect the virus titers in the supernatant. The data were presented as means ±SD with three independent experiments, ** *p* < 0.01. (**F**) DF-1 cells infected with 0.1 MOI of different IBDV strains, including two plasmids recued, four plasmids rescued, and parental IBDV at 12 h, 18 h, 24 h, and 48 h. Collecting the whole cells for TCID_50_ to detect the virus titers in the supernatant.

According to the result of Figure 2, the production of infectious IBDV needs a complete minus-sense or plus-sense segment A/B as well as an RNP complex (VP1 and VP3). Four plasmids transfected into 293T cells could generate infectious IBDV. Next, we sought to integrate the four plasmids into two plasmids (also named as dual-direction promoter, pCMV-mA, and pCMV-mB) by using a polymerase II promoter (Poly II Pro) in the reverse direction (Figure 3A). In the model of Figure 3A, we proposed Poly I Pro transcripts minus-sense segment A/B in the forward direction; meanwhile, Poly II Pro promoted transcription and translation of plus-sense segment A/B with capped mRNA. Then, translated VP1 and VP3 are both recruited to copy minus-sense segment A/B, subsequently to translate viral proteins, and ultimately to generate infectious IBDV. Next, we first assess whether the dual-direction promoter plasmids translate viral proteins. pCMV-mA and pCMV-mB or other indicated plasmids were individually transfected into 293T cells for 72 h. The cells were subjected to western blot. We found that pCMV-mA translates VP2 and VP3 (Figure 3B, lane 2), pCMV-mA translates VP1 (Figure 3B, lane 4), pUC-mA and pUC-mB together with VP1 and VP3 translate VP1–VP3 (Figure 3B, lane 5), indicating that a dual-direction promoter might transcript minus-sense dsRNA as well as translate viral proteins. Thus, we further evaluated whether the dual-direction promoter plasmids produced infectious IBDV. The indicated plasmids were transfected into 293T cells for 72 h. The supernatant of transfected cells was transferred to fresh monolayer DF-1 cells for another 48 h of culturing. As shown in Figure 3C, the specific CPE was observed in DF-1 cells incubated with the supernatant of the cells co-transfected with pCMV-mA and pCMV-mB (Figure 3Cd) and with pUC-mA and pUC-mB together with both VP1 and VP3 (Figure 3Cc) but not in cells co-transfected with other plasmids (Figure 3Ca,b), indicating that integrated dual-direction promoter plasmids can generate infectious IBDV. DF-1 cells infected with the indicated supernatant could produce viral proteins VP1–VP3 (Figure 3D). Rescuing IBDV needs four plasmids, according to the result of Figure 2, but a dual-direction promoter rescue system includes just two plasmids that lead to a high transfection efficiency, which might ultimately result in a high virus rescue efficiency. In fact, we found that virus titers from a rescued dual-direction promoter system were significantly higher than in a rescued four plasmid system (Figure 3E). In addition, a TCID_50_ assay showed that the growth curve of these two rescued IBDV was nearly the same as the parental IBDV (Figure 3F). Taken together, an integrated dual-direction promoter system improves the IBDV rescuing efficiency, which may be due to enhancing transfection efficiency.

### 3.4. Arg186 Monomo-Methylation Facilitates VP1 Activity

**Figure 4:**
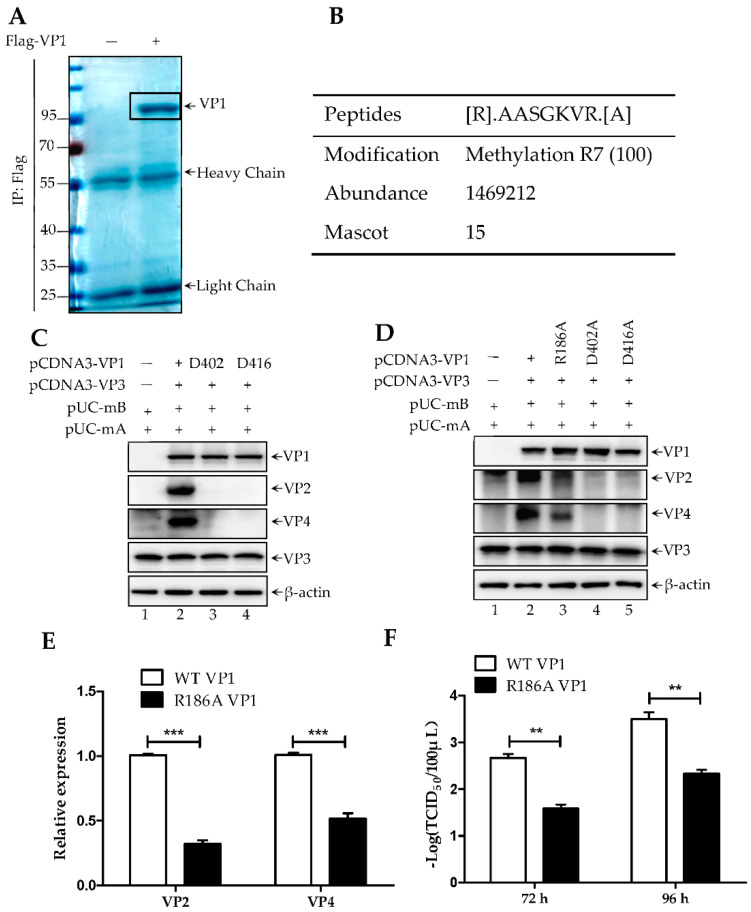
Arg 186 monomer-methylation facilitates VP1 activity. (**A**) 293T cells was transfected with Flag-VP1 (non-transfection as negative control) for 48 h. Whole cell lysates were subjected to immunoprecipitation by using anti-Flag agarose. Purified Flag-VP1 was separate by SDS-PAGE and visualized via Coomassie brilliant blue staining. Specific band of Flag-VP1 was cut for LC–MS. The Arg186 was identified as a monomer-methylation site of which the peptide was shown in the right box (**B**). (**C**) Indicated plasmids were transfected into 293T cells for 72 h, which were furtherly examined by western blotting, using indicated antibodies. (**D**) Indicated plasmids were transfected into 293T cells for 72 h, which were furtherly examined by western blotting, using indicated antibodies. β-actin was used as the loading control. (**E**) The data of band density shown in (**D**) was presented as means ± SD with three independent experiments, *** *p*< 0.001. (**D**) Indicated plasmids were transfected into 293T cells for 72 h and 96 h. The supernatant of indicated transfected cells were subjected to analyze the virus titers using a TCID_50_ assay. The data of virus titers was presented as means ±SD with three independent experiments, ** *p* < 0.01.

Next, we sought to use this dual-direction promoter rescue system to investigate the function of VP1 arginine methylation. First, Flag-VP1 was transfected into 293T cells for 48 h and then subjected to purify by anti-Flag agarose. Purified Flag-VP1 was subsequently used to perform the LC–MS to identify the arginine methylation site (Figure 4A). The result shown in Figure 4B reveals that Arg186 (R186) is a monomer-methylation site with a high confidence. Since VP1 is the polymerase protein of IBDV, we next investigated the role of R186 methylation on polymerase activity. The pUC-mA together with a VP1 and a VP3 co-transfection was used as the polymerase activity detection tool since pUC-mA could be translated to another viral protein, including VP2 and VP4 under co-transfection of both VP1 and VP3 (Figure 4C, lane 2), rather than in co-transfection of D402A VP1 or D416A VP1 and VP3 (Figure 4C, lane 3 and lane 4). Thus, VP2 and VP4 protein levels could present the polymerase activity of VP1. As shown in Figure 4D, the expression level of both VP2 and VP4 was remarkably decreased with a reduction of 40% and 50%, respectively, in the co-transfection of R186A VP1 (Figure 4D, lane 3), compared with the co-transfection of wild type (WT) VP1 (Figure 4D, lane 2), indicating R186 monomer-methylation is critical for VP1 activity (Figure 4E). In addition, we believe that polymerase activity is important for IBDV rescue efficiency. Thus, we attempted to analyze the effect of R186A mutant VP1 on virus rescue efficiency. pCMV-mA and pCMV-mB or pCMV-mB R186A were co-transfected into 293T cells for 72 h and 96 h. The supernatant of transfected cells was subjected to assess the viral titers using TCID_50_ assays (Figure 4F). We found that R186A mutant VP1 severely disturbed the efficient of IBDV rescue. Taken together, a novel polymerase detection tool was generated and revealed that R186, one of arginine monomer-methylation site in the N-terminus of VP1, is essential for VP1 activity.

### 3.5. Mutation of Arg186 Damages IBDV Proliferation Ability

**Figure 5:**
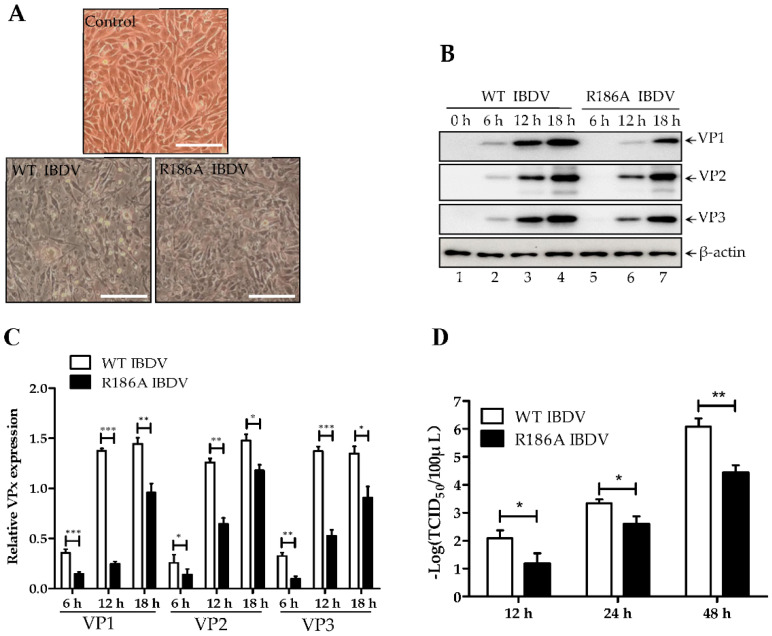
Mutation of Arg 186 damages IBDV proliferation ability. (**A**) The supernatant of 293T cells co-transfected with indicated plasmids was passaged in DF-1 cells at 48 h post-transfection, and the images of CPE were taken under a phase-contrast microcopy at 48 h post-infection. Scale bar, 100 μm. (**B**) DF-1 cells infected with 0.1 MOI indicated IBDV at 6 h, 12 h, and 18 h were analyzed by western blot using VP1/VP2/VP3 antibodies. (**C**) Comparison of the expression of VP1/VP2/VP3 in different lanes in (**B**). The density of VP1/VP2/VP3 and β-actin were quantified by Image J. Data were presented as means ± SD. * *p* < 0.5; ** *p* < 0.01; *** *p* < 0.01. (**D**) Monolayer DF-1 cells (triplicates in 12-well plate) were infected with WT IBDV or R186A IBDV at 0.1 MOI at 12 h, 24 h, and 48 h; the virus titers in the supernatant were determined by TCID_50_ assay. Data were presented as means± SD. ns, not significant; * *p* < 0.5; ** *p* < 0.01.

Since R186A methylation mutation severely damages VP1 activity, the role of R186 methylation in IBDV proliferation is not clear. Thus, a dual-direction promoter rescue system was used to produce VP1R186A mutant IBDV. pCMV-mA and pCMV-mB or pCMV-mBR186A mutant were co-transfected into 293T cells for 72 h. The fresh DF-1 cells incubated with the supernatant of indicated transfected cells were furtherly cultured for another 72 h. We found that VP1R186A mutant IBDV was rescued successfully with observation of CPE that was also induced by WT IBDV (Figure 5A). The expression of all viral proteins, including VP1–VP3, of R186A mutant IBDV were significantly less than that of WT IBDV at different times post-infection with 0.1 MOI IBDV (Figure 5B,C), suggesting the R186A mutant might disturb IBDV replication. To further confirm the role of VP1 R186 methylation in the IBDV life cycle, we performed the growth curve determination; equally, 0.1 MOI WT IBDV or VP1R186A IBDV were individually replicated in DF-1 cells at different times. The whole cells were collected for a TCID_50_ assay, which showed that viral titers of WT IBDV were remarkable higher than those that were VP1R186A mutant IBDV, with an increase of 10–20 fold at 12–24 h post-infection. These data thus suggest that R186 methylation in the N-terminus VP1 facilitates IBDV proliferation.

## 4. Discussion

In this study, we generated a dual-direction promoter, reverse genetic system with rapid, high efficiency IBDV rescue. The dual-direction promoter, reverse genetic system was first performed to produce the avian influenza virus (AIV) [20]. The genome of AIV contains eight minus-sense RNA that need eight plasmids co-transfecting into cells, which leads to low rescue efficiency due to low transfection efficiency. However, the IBDV genome contains two segments of dsRNA. Thus, we used this promoter to transcript the minus-sense IBDV genome and then rescue IBDV together with VP1 and VP3. To improve virus rescue efficiency, we engineered the polymerase II promoter (Poly II Pro, CMV promoter) and Poly I Pro in the reverse direction. The CMV promoter was inserted into the end of Terminator I to support the transcription of plus-sense RNA sequence (reverse direction) to produce mRNA that are directly translated into viral proteins VP1-VP5. Poly I Pro transcripts minus-sense RNA, which requires both VP1 and VP3 to copy into plus-sense RNA and then translate into viral proteins, ultimately generating infectious IBDV. Nevertheless, with the integration of both Poly I Pro and Poly II Pro, we do not need to make the VP1 and VP3 accessory plasmids. Critically, we found that the efficiency of IBDV rescue through transfection of two plasmids was remarkably higher than that through transfection of four plasmids, possibly due to improving transfection efficiency. However, since IBDV replicates more efficiently in avian cell lines (such as DF-1 cells) than in human cell lines, if the first step of IBDV rescue was performed in DF-1 cells, it may furtherly improve rescue efficiency. In 1999, both T7 and CMV promoters were used for the first time to generate mutant IBDV efficiently within 4 days [21]. Subsequently, in 2007, an IBDV rescue system was established based on two plasmids using the CMV promoter together with hammerhead ribozyme (HamRz) and hepatitis delta ribozyme (HdvRz), which have to be passaged three times in DF-1 cells [22]. In addition, once IBDV rescue operations were performed in DF-1 cells, the human Poly I Pro requires to be placed by avian Poly I Pro to facilitate transcript efficiency. Altogether, our work expanded a new method for rescuing IBDV or other dsRNA virus members.

In addition, we also used the Poly I Pro driven minus-sense segment A plasmid as a polymerase activity analyzing tool for detecting VP2 and VP4, the major protein translated by segment A. Our data showed that the VP activity dead mutant D402A and D416A cannot promote Poly I Pro driven minus-sense segment A plasmid translation of VP2 and VP4, even together in a co-transfection with VP3. Previous reports suggested that several mini-genome reporter systems (such as EGFP or Luciferase) are commonly used to analyze polymerase activity, such as the Ebola virus or AIV [23,24,25,26,27,28]; however, these minigenome reporter genes used in IBDV polymerase activity analysis cannot exclude background signals, resulting in a decrease in the sensitivity of polymerase activity detection. Therefore, we developed this system to analyze VP1 activity, which worked efficiently.

Arginine methylation, a classified protein post-translational modification, is mediated by a member protein arginine methyltransferase, which furtherly regulates multiple signaling pathways [29,30]. Recently, it has been suggested that arginine methylation plays a critical role in regulating the replication of many viruses [31,32]. The VP1 N-terminal domain (residues 1–168) surrounds the central polymerase domain, which bridges the fingers and the thumb on one side of the catalytic clef, indicating that the N-terminus is critical for VP1 activity [33,34]. LC–MS together with mutations showed that the Arg186 is an arginine monomer-methylation site. To obtain Arg186 mutant IBDV, the dual-direct promoter rescue system was used to produce mutant IBDV. Our data indicated that Arg186 mutations severely disrupted VP1 activity as well as viral proliferative ability and IBDV could hijack the host Arg methylation system to attack VP1, ultimately promoting IBDV replication, although the methyltransferase was not precisely identified here.

## 5. Conclusions

The established IBDV dual-direction promoter-rescue system has high efficiency in rescuing infectious IBDV. It may take great candidate usage to provide an efficiency tool for modifying IBDV. Furthermore, the results of LC–MS revealed that Arg 186 is the arginine monomer-methylation site, indicating that VP1 is an arginine methylation protein. In addition, mutation of Arg 186 severely damaged VP1 activity and IBDV replication ability, but the detailed molecular of VP1 arginine methylation requires further exploration in the future studies.

## Data Availability

All the data analyzed in this study are available from the corresponding author.
